# Global Transcriptomic Analysis of Human Neuroblastoma Cells in Response to Enterovirus Type 71 Infection

**DOI:** 10.1371/journal.pone.0065948

**Published:** 2013-07-05

**Authors:** Li-Juan Xu, Tao Jiang, Fu-Jun Zhang, Jian-Feng Han, Juan Liu, Hui Zhao, Xiao-Feng Li, Rui-Ju Liu, Yong-Qiang Deng, Xiao-Yan Wu, Shun-Ya Zhu, E-De Qin, Cheng-Feng Qin

**Affiliations:** 1 Department of Virology, State Key Laboratory of Pathogen and Biosecurity, Beijing Institute of Microbiology and Epidemiology, Beijing, China; 2 PLA 404 Hospital, Weihai, China; 3 Rizhao Hospital of Traditional Chinese Medicine, Rizhao, China; 4 Liaocheng People’s Hospital, Liaocheng, China; Johns Hopkins School of Public Health, United States of America

## Abstract

Human enterovirus type 71 (EV71) is the major pathogen of hand-foot-and-mouth disease (HFMD) and has been associated with severe neurological disease and even death in infants and young children. The pathogenesis of EV71 infection in the human central nervous system remains unclear. In this study, human whole genome microarray was employed to perform transcriptome profiling in SH-SY5Y human neuroblastoma cells infected with EV71. The results indicated that EV71 infection lead to altered expression of 161 human mRNAs, including 74 up-regulated genes and 87 down-regulated genes. Bioinformatics analysis indicated the possible roles of the differentially regulated mRNAs in selected pathways, including cell cycle/proliferation, apoptosis, and cytokine/chemokine responses. Finally, the microarray results were validated using real-time RT-PCR with high identity. Overall, our results provided fundamental information regarding the host response to EV71 infection in human neuroblastoma cells, and this finding will help explain the pathogenesis of EV71 infection and virus-host interaction.

## Introduction

Human enterovirus 71 (EV71) is a single-stranded, positive-sense RNA virus that belongs to the genus *Enterovirus*, family *Picornaviridae* [1]. EV71 has been well recognized as the major pathogen that causes hand-foot-and-mouth disease (HFMD) in infants and young children. Since 1997, large HFMD epidemics with severe neurological complications were associated with EV71 infection, and fatalities were frequently reported in the Asian-Pacific region [2], [3]. In China, a large-scale HFMD outbreak gave rise to 2,203,597 reported cases that caused 559 deaths in 2012 (http://www.moh.gov.cn/mohjbyfkzj/s6873/list.shtml). Currently, there is neither an approved vaccine nor any effective antiviral drugs for EV71 infection.

Until now, the pathogenesis of EV71 infection has remained unclear. The clinical significance and major cause of death of EV71 were due to severe neurological complications, including extensive neuronal degeneration, central nervous system (CNS) inflammation and pulmonary congestion with hemorrhage. Recently, EV71 infection with CNS involvement and cardiopulmonary failure were associated with neurologic sequelae, delayed neurodevelopment, and reduced cognitive functioning [2]. Mouse experiments revealed that EV71 invaded the CNS through retrograde axonal transport and that hematogenous transport represented only a minor route of transmission [4], [5], [6]. Histopathological changes, such as neuronal degeneration, neuronal loss and neuronophagia, were observed in the spinal cord, brainstem, and skeletal muscle, along with necrotizing myositis and splenic atrophy, after gerbils were inoculated intraperitoneally with EV71 [7]. Additionally, radiological or histopathological evidence of the induction of paralysis, a similar process to that of poliovirus, was also observed during EV71 infection [8], [9], [10], [11]. Post-mortem studies revealed EV71-associated neurogenic pulmonary edema [12], [13], [14], [15], which was in agreement with the experimental infection of cynomolgus macaques with EV71 [16], [17], [18], [19].

The host response to viral infection represents complex and divergent pathways that interact with the invading viruses. Systemic analysis of the host response to EV71 infection in human neural cells will provide critical clues to understand the molecular mechanisms of EV71-associated neurological complications. In terms of adaptability and computational resources, microarray technology makes it possible to assess the expression profiles for many non-model species. Microarrays have been used to analyze global gene expression at both the cell culture and organismal levels for many viruses, including dengue virus, Epstein-Barr virus, human immunodeficiency virus, avian influenza A (H5N1), and human hepatitis C virus, etc. [20], [21], [22], [23], [24]. Recently, Leong et al. analyzed host gene expression in EV71-infected RD cells, EV71-permissive human muscle cells, using microarray analysis [25]. Human glioblastoma SF268 cells were also used to study global changes in mRNA expression during EV71 infection, and the results indicated that genes associated with chemokines, interferon, complement activation and apoptosis were up-regulated [26].

In this study, gene expression profiling data from human neuroblastoma SH-SY5Y cells infected with EV71 was obtained using microarray analysis and validated using real-time RT-PCR. Bioinformatics analysis revealed that the differentially regulated mRNAs were associated with cell cycle/proliferation, apoptosis and cytokine/chemokine responses in the host.

## Materials and Methods

### Cells and Viruses

Human neuroblastoma cells SH-SY5Y (ATCC, CRL-2266™) was cultured in Dulbecco's modified Eagle's medium/F-12 (DMEM/F-12, Invitrogen), supplemented with 10% fetal bovine serum (FBS) (Gibco), 50 U/ml penicillin and 0.1 mg/ml streptomycin at 37°C in 5% CO_2_. Human rhabdomyosarcoma (RD, ATCC, CCL-136™) cells were grown in DMEM supplemented with 10% FBS. The EV71 strain AH08/06, classified as EV71 subtype C4, was isolated from a throat swab sample of an HFMD case during an outbreak in 2008 in Anhui, China [27]. The viral stocks were prepared in RD cells, and virus titers were determined using a plaque-forming assay as previously described [28].

### Viral Growth Kinetics in SH-SY5Y Cells

SH-SY5Y cells were infected with EV71 at a multiplicity of infection (m.o.i.) of 1 and incubated at 37°C for 2 h. The infected cells were washed twice with phosphate-buffered saline (PBS) and refed with fresh medium. Viral cultures were harvested at 6, 12, 24, 48 and 72 h post-infection (h.p.i.), and the virus titer was determined using a standard plaque-forming assay on RD cells as previously described [28]. The plaque assays were carried out in triplicate.

An indirect immunofluorescence assay (IFA) was performed as previously described [29]. Briefly, SH-SY5Y cells were infected with EV71 strain AH08/06 and fixed at 24 h.p.i. The cells were incubated with EV71 monoclonal antibody EVF12 and then treated with a 200-fold dilution of ﬂuorescein isothiocyanate (FITC)-labeled anti-globulin (Chemicon) in 0.02% (w/v) Evans blue. After multiple washes, the positive cells were detected using a fluorescent microscope (OLYMPUS, Tokyo, Japan).

### Microarray Analysis

SH-SY5Y cells (2×10^5 ^cell/ml) were seeded onto 6-well plates and then incubation infected with EV71 at 1 m.o.i. overnight. After incubation, the cells were washed with PBS and refed with a 2% containing medium. Cells were treated with TRIzol (Invitrogen) and frozen for microarray analysis of their genetic expression profile. Total RNAs were extracted using the NucleoSpin® RNA clean-up kit (MACHEREY-NAGEL, Germany) at 12 h.p.i.

To analyze the host gene expression, the 35K Human Genome Array (Operon) comprising oligonucleotide probes that average at 70 bases in length for 35035 genes from the human genome oligo database (human_V4.0) was used. Total RNA was reverse transcribed using the Cbc Script II reverse transcriptase and T7 oligo (dT) primer, substituting a fraction of the dTTP in the newly synthesized strands with aminoallyl-dUTP (AA-dUTP). Dye-labeled cDNA was created using Cy3 dye and Cy5 dye (GE Healthcare Cat. No. PA55021/PA53021) according to the manufacturer’s protocols. Hybridization to the microarray was performed according to the manufacturer’s protocol of SmartArray™ (CapitalBio Corp., Beijing, China). All of the data were submitted to the GEO microarray database according to the LuxScan 3.0 standards (CapitalBio). All of the files were transformed and normalized using Loess normalization techniques. A two-way analysis of variance (ANOVA) was performed (infection and control) (p<0.05). The degree of fold-change (relative fluorescent intensity) was analyzed for all of the differentially regulated genes using the SAM software [30]. A list of significant genes was generated and a hierarchical clustering was performed. The microarray analysis dataset was submitted to Gene Expression Omnibus under the accession number GSE45589.

### Ontology (GO) Terms and KEGG Pathway Annotation

For the statistical analyses, the genes that were significantly up- or down-regulated (p<0.05) with ratios >2 or <0.5 were selected and included in the database for modeling into ontological networks (CapitalBio® Molecule Annotation System, V3.0). Network modeling was then performed to determine the interactions between the significant genes, canonical pathways analysis to determine the genes involved in known pathways and disease/physiological function/location annotation using Fisher’s exact test. This process was performed on all of the significant genes and on the gene ontology enriched datasets.

### Confirmation of Microarray Data for Selected Genes by Real-time RT-PCR

A total of twelve genes of interest (p<0.05, fold change >2 or <0.5) were selected for further validation using SYBR green-based real-time RT-PCR.

Real-time RT-PCR primers were designed and synthesized using Beacon Designer software (Table 1); GAPDH was set to be the endogenous control. Briefly, each 20 µl reaction included 2 µl of total RNA, 0.4 µM of each primer, 1.2 µl TaKaRa Ex Taq HS Mix, 0.4 µl PrimeScript PLUS RTase Mix, and 10 µl of 2×One step SYBR RT-PCR Buffer 4 (Roche). The reactions were subjected to reverse-transcription of 42°C 5 min and 95°C for 10 s, followed by 40 cycles each of 95°C for 5 s, 60°C for 20 s, and subjected to melting curve analysis. Each gene was quantified relative to the calibrator. Calculations were made using the instruments and equation 2^−ΔΔCT^. Each assay was performed in triplicate.

**Table 1:** Primers used for Real-time RT-PCR analysis.

Gene	Primer sequence (5′ →3′)	
	Sense	Antisense
**SMC1A**	GCTACAAGACCTGAAGAATC	CGAATCTCCTCCATCTCAT
**SMARCC1**	GCTACAAGACCTGAAGAATC	ACTTCTGACTGGCTCTTC
**SGK**	CAAGACACAAGGCAGAAGA	CATTCCGCTCCGACATAA
**CYFIP1**	GTCTGTACCTGATGGATGG	CCTGGAGTTGCTTGAAGT
**KIAA1212**	TGCTACAGAACAGAACAC	GTAATCCAGTTGCCTCTC
**AMOTL2**	AGAACAACTGCGAGAGAA	AAATACTTCTGCTCCCACTT
**PRKAA2**	ATAGACAGAAGATTCGCAGTT	CCTCCAGACACATATTCCAT
**NDEL1**	GCTGATAACCAAAGACTGAA	AACACTGAGACCTGCTTA
**PCYT2**	CTCACCACAGACCTCATC	TCCTTGGCTTCCTTCTTC
**TCN1**	TCAGGTAACTTCAACATCTC	TTAGCACAGTGACATTGG
**SF3B3**	CTTGTCCTGCTCCTTATTAG	CTTGTCTGCTCGTATGTG
**BCAN**	GGAAGGTAAGGCATTGGA	CCTCCTCCTCTTCTTCTTC
**GAPDH** *	AGAAGGCTGGGGCTCATTTG	AGGGGCCATCCACAGTCTTC

* Housekeeping gene transcript serving as the normalization control for real-time RT-PCR.

Other genes were selected from the microarray analysis for real-time RT-PCR.

## Results

### Human Neuroblastoma Cells are Permissive to EV71 Infection

**Figure 1:**
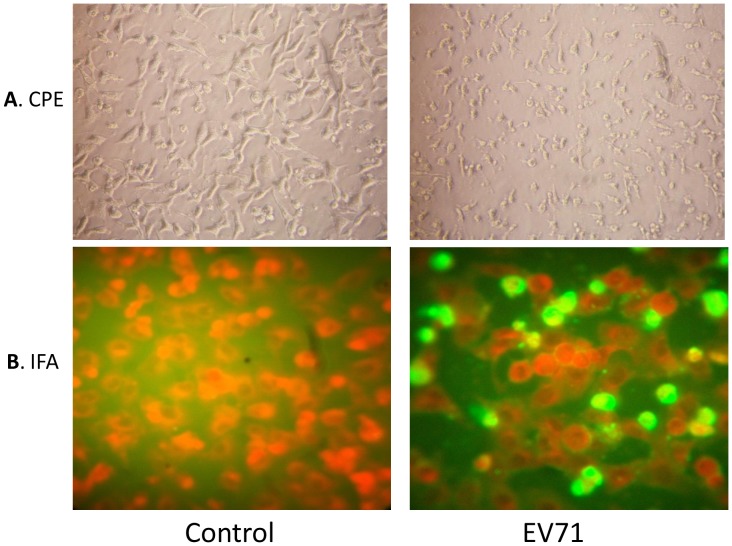
Human SH-SY5Y cells are permissive to EV71 infection. A. Typical CPE caused by EV71. Cells infected with EV71 were observed and photographed using an inverted microscope (Olympus, 40×) at 48 hours post-infection. The uninfected cells were shown in parallel as control (mock). B. Viral protein expression in SH-SY5Y cells infected with EV71. The viral protein was detectable after the cells infected with EV71 24 h by IFA assay (OLYMPUS, Tokyo, Japan).

**Figure 2:**
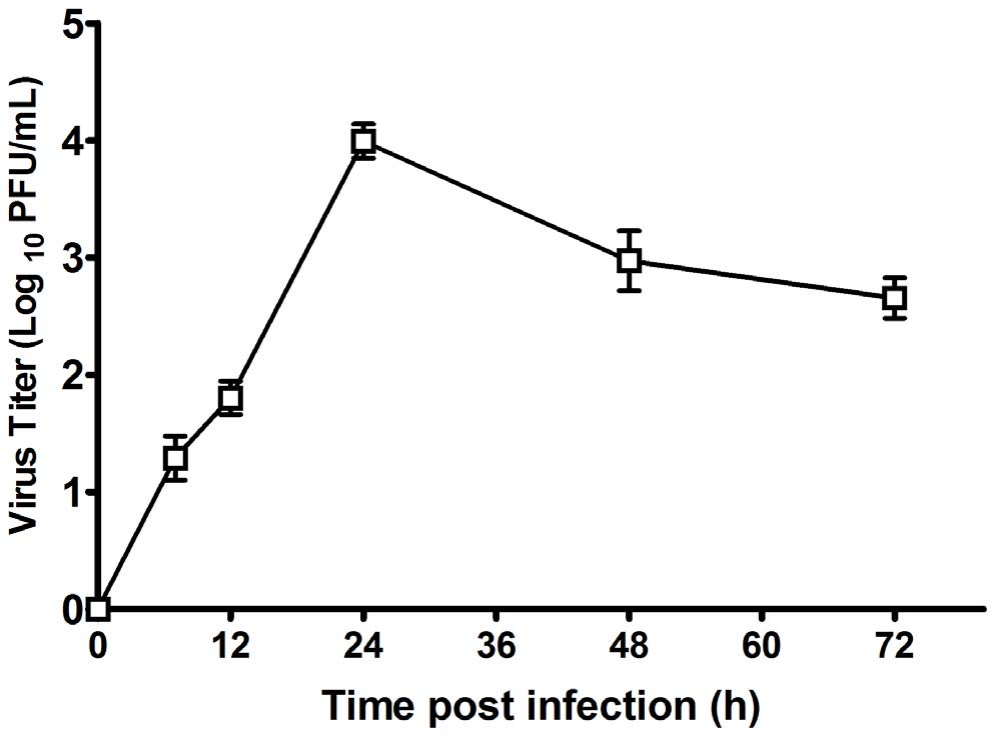
One-step growth curves of EV71 strain AH/08/06 in SH-SY5Y cell lines. SH-SY5Y cells were inoculated with the EV71 virus, and supernatant cultures were collected at 6, 12, 24, 48, and 72 hours post-infection and virus titers were determined using a plaque assay.

In this study, to ensure that human neuroblastoma cells could be used for microarray analysis, SH-SY5Y cells were infected with EV71 at an m.o.i. of 1. As shown in Figure 1A, EV71 infection in SH-SY5Y cells caused significant cytopathic effects (CPE), including rounding up, aggregation, and death, at 48 h.p.i. The viral protein was also detectable in both the nucleus and cytoplasm of the SH-SY5Y cells by IFA assay (Figure 1B). One-step viral growth curve assay indicated that EV71 replicated efficiently in SH-SY5Y cells and peaked at approximately 10^4 ^PFU/ml at 24 h.p.i. (Figure 2). This result showed that human neuroblastoma SH-SH5Y cells are permissive to EV71 infection in vitro.

### Global Transcriptomic Analysis of EV71-infected SH-SY5Y Cells

**Figure 3:**
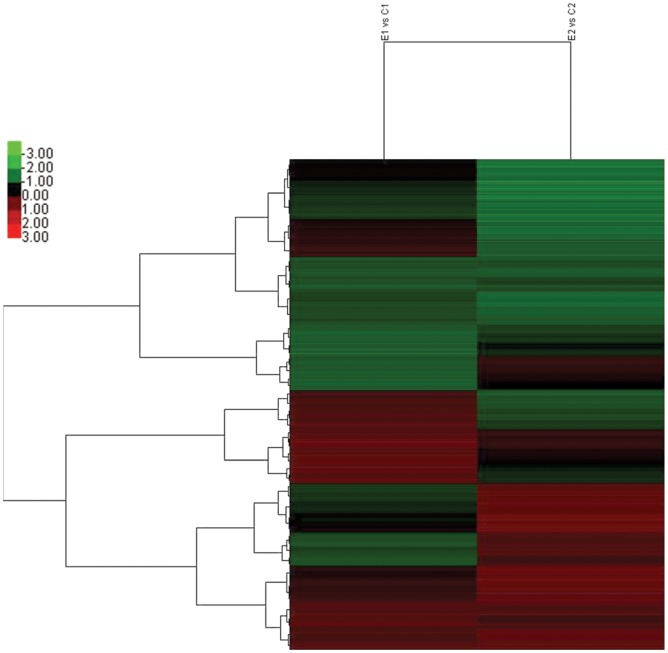
Heat map of the microarray gene expression profile of SH-SY5Y cells infected with EV71. The two-way hierarchical cluster heat map showed selected significantly expressed mRNAs in two independent samples. Each row shows the relative expression level for a single mRNA, and each column shows the expression level of a single sample. The mRNAs were chosen according to the cutoff p<0.01 in direct comparison. Red represents mRNAs with increased expression, and green represents mRNAs with decreased expression.

To perform whole human genomic array analysis, SH-SY5Y cells infected with EV71 were subjected to 35K Human Genome Array. The differentially transcribed genes in SH-SY5Y in response to EV71 infection were shown in clustering analysis (Figure 3). Microarray hybridization preliminarily identified 161 genes as being differentially expressed during EV71 infection, including 74 up-regulated genes (Table S1) and 87 down-regulated genes (Table S2). Some of these genes have been identified in association with neurological disorders and may therefore have roles in the nervous system lesion-induced EV71 infection, such as *SGK*, *CYFIP1*, *NDEL1*, *KIAA1212*, *BCAN*, *SF3B3*, *SMC1A*, and *TCN1*. Other genes, such as *AMOTL2*, *PCYT2*, and *SMARCC1,* were reported to relate to immune and inflammatory responses.

**Figure 4:**
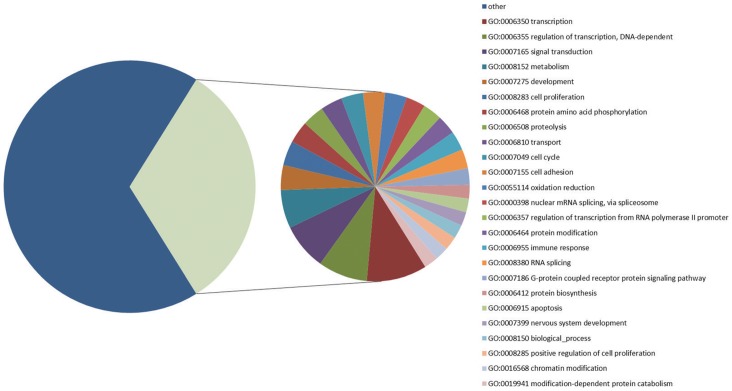
Enriched GO terms in the biological process category among the differentially expressed genes. Enriched GO analysis of the microarray data was performed using the web-based analysis software CapitalBio® Molecule Annotation System V3.0. The results indicated the biological processes performed by the differentially expressed genes (p<0.01) in the EV71-infected SH-SY5Y cells. Each section represents a different type of biological process. The number of genes enriched is shown after the name of the process.

To facilitate the analysis of our data, the on-line CapitalBio® Molecule Annotation System V3.0 was used to identify the gene ontology functional classes and pathways that were enriched among the differentially expressed genes in EV71-infected SH-SY5Y cells compared with the non-infected cells. Firstly, significantly up-regulated (ratio >2) or down-regulated (ratio <0.5) (p<0.05) genes were selected and included in the database for modeling into ontological networks for GO analysis. The results showed that each section represented a different type of biological process found in EV71-infected SH-SY5Y cells, including transcription, cell proliferation, metabolism, immune responses (Figure 4). Then, the significantly expressed genes (P<0.001) were assigned in the KEGG pathway, as shown in Table 2. The significant P-values determine the probability of the association between the genes in the dataset and the KEGG pathway. Regulation pathways, such as the Notch signaling pathway, cell cycle, p53 signaling pathway, which play critical roles in cell proliferation, apoptosis and growth, respectively, were identified during EV71 infection in SH-SY5Y cells (Table 2).

**Table 2:** Analysis pathway of different globle genes by KEGG.

Pathway name	Count*	p-Value
Retinol metabolism	12	3.37E-18
Drug metabolism - other enzymes	11	1.36E-17
Pentose and glucuronateinterconversions	9	6.63E-17
Ascorbate and aldarate metabolism	9	1.01E-16
Porphyrin and chlorophyll metabolism	9	1.09E-14
Androgen and estrogen metabolism	9	2.72E-14
Metabolism of xenobiotics by cytochrome P450	10	3.51E-14
Drug metabolism - cytochrome P450	10	4.71E-14
Starch and sucrose metabolism	9	1.11E-13
Cytokine-cytokine receptor interaction	8	2.69E-06
Hematopoietic cell lineage	4	1.99E-04
Notch signaling pathway	3	5.02E-04
Cell cycle	4	6.55E-04
Methionine metabolism	2	0.002576
Nitrogen metabolism	2	0.0028042
Calcium signaling pathway	4	0.0032365
Aminoacyl-tRNA biosynthesis	2	0.0080336
Fatty acid metabolism	2	0.0096166
Cell adhesion molecules (CAMs)	3	0.0098716
Jak-STAT signaling pathway	3	0.0145845
p53 signaling pathway	2	0.0216548
PPAR signaling pathway	2	0.0222445
Chronic myeloid leukemia	2	0.0252913
Ribosome	2	0.0443484
Toll-like receptor signaling pathway	2	0.0443484
T cell receptor signaling pathway	2	0.0499375

* The number of genes in each pathway.

### Validation Using Real-time RT-PCR

**Figure 5:**
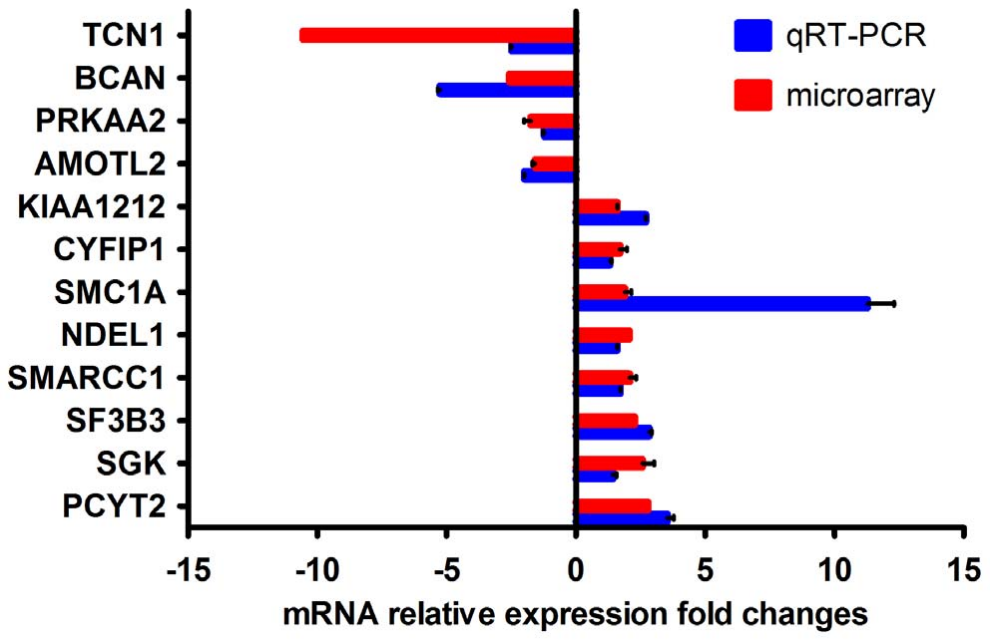
Valuation differential expression selected genes by real-time RT-PCR. Twelve expression levels of selected genes from the microarray assay were validated by real-time RT-PCR at 12 h.p.i EV71 infection. The relative fold change was calculated based on an endogenous control normalization and repeated three times independently.

To confirm the microarray results, the relative abundances of the selected mRNAs were assayed using a real-time RT-PCR assay. Twelve cellular genes (Table 1) associated with neurologic sequelae were selected for further validation. The fold changes of mRNAs in the EV71-infected SH-SY5Y cells were calculated using instruments and equation 2^−ΔΔCT^ (Figure 5). Real-time RT-PCR revealed that the mRNA expression level of *SMC1A* was up-regulated 11-fold at 12 h.p.i. In addition, the mRNA expression level of *TCN1* was down-regulated 10-fold at 12 h.p.i. according to qRT-PCR. The fold-change calculated by qRT-PCR for *SMC1A* and *TCN1* was greater than that suggested by the microarray data. This finding is in accordance with comparative analysis, which observed that fold change results determined by qRT-PCR were greater than those determined by microarray analysis [31], [32]. However, the obtained expression patterns from microarray data and qRT-PCR showed the same directions of response in both methodologies, as shown in Figure 5, indicating the high quality and reliability of the microarray data analysis.

## Discussion

In this study, to better understand the molecular mechanism of EV71 infection in human neuroblastoma cells, the responses of SH-SY5Y cells to EV71 infection were analyzed using microarray and validated using real-time RT-PCR. According to the global mRNA profile analysis, 161 differentially regulated genes, including 74 up-regulated genes and 87 down-regulated genes, were classified according to their functional roles during EV71 infection. Bioinformatics analysis indicated that the differentially regulated mRNAs were associated with host cellular pathways involved in cell cycle/mitosis/proliferation/apoptosis, cytokine/chemokine and immune responses. Previously, mRNA profiling in EV71-infected RD cells indicated that transcription and translation regulators were suppressed and that apoptosis genes were up-regulated [25]. Microarray analysis for EV71-infected SF268 cells revealed that the up-regulated genes were associated with chemokines, interferon, complement activation, and apoptosis, and the down-regulated genes were involved in transcription and translation regulators [26]. Global transcriptomic analysis in various cells will help to better understand the host responses to EV71 infection.

SH-SY5Y cells are the third successive subclone of SK-N-SH (human neuroblastoma) cells [33], [34], [35] and have been used to study the pathogenesis of poliovirus [36], [37]. Recently, SH-SY5Y cells have been used to study the EV71 site-specific adaptations an the increased neural cell tropism during EV71 infection [38]. In this study, SH-SH5Y human neuroblastoma cells were found to be permissive to EV71 strain AH/08/06 infection in vitro (Fig. 1A). The AH/08/06 strain is classified as subgenotype C4, which has been the sole viral genetic lineage circulating in the mainland since 2007. The strain used in this study exhibits similar recombination events as many other EV71 isolates in mainland China [39], [40], [41].

According to the microarray data and real-time RT-PCR results, the differences among mRNA expression levels of genes related to the cytoskeleton and motility were significant. The *NDEL1* gene was 2-fold up-regulated in EV71-infected SH-SH5Y cells. NDEL1 is a substrate for the serine/threonine protein kinase CDK5, which is essential for cortical neuronal migration. *NDEL1* is also linked to the etiology of various mental illnesses and neurodegenerative disorders. For example, DISC1, another NDEL1-interacting partner, plays essential roles in neuronal proliferation, neuronal migration and axon guidance and has been implicated in schizophrenia and related psychiatric disorders [42], [43], [44], [45]. The potential roles of neurological diseases during EV71 infection deserve further investigation. Additionally, SF3B3, which is a component of the minor U12-type spliceosome, was 2.8-fold up-regulated in response to EV71 infection. Kotake et al. suggested that a splicing impairment of pladienolide may cause the organism to repress the expression of certain genes essential for cell proliferation or cell survival [46]. In our study, up-regulation of the *SF3B3* gene may be related to SH-SY5Y cell proliferation or cell survival during EV71 infection.

Transcription, pre-mRNA processing (capping, splicing and polyadenylation), mRNA surveillance and mRNA export compose the process of gene expression. These steps are extensively coupled to form gene expression factories. In our results, CYFIP1 (also known as Sra-1), which is a clathrin heavy-chain binding protein associated with mental retardation and specifically expressed in the nervous system, was up-regulated in the SH-SY5Y cells by EV71 infection. The levels of the protein encoded by fragile X mental retardation protein (FMRP), which inhibits translation initiation, and target mRNAs are increased upon the reduction of CYFIP1 in neurons. Moreover, brain cytoplasmic RNA 1 (BC1) increases the affinity of FMRP for the CYFIP1-eIF4E complex. Sukarieh et al. found that the nuclear re-localization of eIF4E corresponds with the repression of host protein synthesis in response to poliovirus infection [47]. It has been demonstrated that picornavirus infection could repress eIF4E expression [48].

EV71 has been demonstrated to induce apoptosis in several cell lines [49], [50], [51]. Apoptosis-associated proteins were regulated to various degrees, notably to support the apoptosis-evasive effects of EV71. In neuronal disease, serum- and glucocorticoid-regulated kinase (SGK) regulates glutamate receptors and up-regulates glutamate transporters. SGK1 participates in diverse biological processes and in the signaling of brain-derived neurotrophic factor (BDNF) and transforming growth factor-b (TGF-b), both of which are neuroprotective after seizures. Schoenebeck et al. found that *SGK1* was up-regulated in various neurotoxic animal models of Parkinson’s disease [52], which may be linked to the CNS complications of EV71 infection. Another apoptosis-associated gene, *FEM1B,* was down-regulated during EV71 infection. Several studies have shown that *FEM1B* induces apoptosis when expression is increased in cancer cells, including breast cancer, cervical cancer, neuroblastoma, and fibrosarcoma cells [52], [53], [54].

The genes encoded a number of cell-signaling proteins involved in immune responses and cell proliferation, such as *SMARCC1*, which was up-regulated during EV71 infection. The protein encoded by *SMARCC1* is a member of the SWI/SNF family, which controls cellular processes, such as growth, development, cell cycle, differentiation, apoptosis, retroviral infection, and carcinogenesis. Jeong et al. indicated that mice that constitutively expressed the SWI/SNF complex in T cells were much more susceptible to experimentally induced autoimmune encephalomyelitis than were normal mice [55]. Additionally, angiomotin-like protein 2 (AMOTL2) and two other members of the motin family are the Yes-associated protein 1 (YAP1)-associated proteins, which regulate the expression of several proliferation- and apoptosis-related genes. It has been reported that AMOTL2 is essential for cell movements in vertebrate embryos [56] and serves as a scaffolding protein to regulate Wnt/β-catenin [7]. In addition, the phosphate cytidylyltransferase 2 (*PCYT2*) gene was 3.5-fold up-regulated during EV71 infection in SH-SY5Y cells. The formation of CDP-ethanolamine is catalyzed by PCYT2 in the Kennedy pathway of phospholipid synthesis and is involved in cell signaling, cell division, and apoptosis. Furthermore, their study suggested that both EGR1 and NF-κB could be potential regulators of *PCYT2* gene transcription [57].

Overall, bioinformatics analysis of EV71 infection showed that differentially regulated mRNAs were associated with the host cellular pathways directing cell cycle/proliferation, apoptosis and cytokine/chemokine and immune responses. This finding requires further laboratory and clinical analysis, including assays to confirm the changes in protein levels. The expression of up/down-regulated genes, such as *PCYT2*, *SGK*, *SMC1A*, *SMARCC1*, *BCAN* and *TCN1*, in EV71-infected SH-SY5Y cells also warrants further study to verify whether they act directly or indirectly to initiate the neurologic sequelae. Regardless of the answer, this study provided important information on the host response to EV71 infection, which lays the foundation to understand the pathophysiological mechanisms of EV71 infection in human neural cells and the CNS.

## Supporting Information

Table S1 **Up-regulated genes in EV71-infected SH-SY5Y cells.**
(DOC)

Table S2 **Down-regulated genes in EV71-infected SH-SY5Y cells.**
(DOC)
