# Supplementary material for: Global Transcriptomic Analysis of Human Neuroblastoma Cells in Response to Enterovirus Type 71 Infection
Source: PLoS One. 2013 Jul 5;8(7):e65948. doi: 10.1371/journal.pone.0065948 (PMC3702535; doi:10.1371/journal.pone.0065948)
Supplement: Table S1 — Up-regulated genes in EV71-infected SH-SY5Y cells. (DOC) [file pone.0065948.s001.doc]

**Table S1. Up-regulated genes in EV71-infected SH-SY5Y cells.**

| **Symbol** | **Description** | **Gene Bank** | **Fold change** |
| --- | --- | --- | --- |
| **ING1** | Inhibitor of growth protein 1 | NM_198219 | 6.2891 |
| **STRA13** | stimulated by retinoic acid 13 | NM_144998 | 5.4677 |
| **MAN2C1** | Alpha-mannosidase2C1 | NM_006715 | 4.1699 |
| **STEAP2** | Six-transmembrane epithelial antigen ofprostate 2 | NM_152999 | 3.7662 |
| **HHAT** | Protein-cysteine N-palmitoyltransferase HHAT | NM_018194 | 3.477 |
| **KLF15** | Krueppel-like factor 15 | NM_014079 | 3.4123 |
| **1-Mar** | membrane-associated RING-CH protein I | NM_017923 | 3.2856 |
| **VAMP3** | Vesicle-associated membrane protein 3 | NM_004781 | 3.1032 |
| **OGFR** | Opioid growth factor receptor | NM_007346 | 3.0391 |
| **SGK** | **Serine/threonine-protein kinase Sgk1** | **NM_005627** | **2.8922** |
| **MACF1** | "Microtubule-actin cross-linking factor 1, isoforms 1/2/3/5 | NM_012090 | 2.855 |
| **PCYT2** | **phosphatecytidylyltransferase** | **NM_002861** | **2.7834** |
| **TRDMT1** | tRNA (cytosine-5-)-methyltransferase | NM_176084 | 2.7797 |
| **PPP4R2** | "protein phosphatase 4, regulatory subunit 2 | NM_174907 | 2.7276 |
| **EIF4H** | Eukaryotic translation initiation factor 4H (eIF-4H) | NM_022170 | 2.6862 |
| **HNRPH2** | Heterogeneous nuclear ribonucleoprotein H' (hnRNP H') (FTP-3) | NM_019597 | 2.6665 |
| **HOMER2** | Homer protein homolog 2 | NM_199330;NM_199331;NM_004839;NM_199332 | 2.6202 |
| **DEFB123** | "Beta-defensin 123 precursor | NM_153324 | 2.616 |
| **CDC25A** | M-phase inducer phosphatase 1 | NM_201567;NM_001789 | 2.6099 |
| **TTC8** | Tetratricopeptide repeat protein 8 | NM_144596;NM_198309;NM_198310 | 2.6068 |
| **STCH** | Stress 70 protein chaperone microsome-associated 60 kDa protein precursor | NM_006948 | 2.5754 |
| **CNTN1** | Contactin-1 precursor | NM_175038;NM_001843 | 2.5612 |
| **QARS** | Glutaminyl-tRNAsynthetase | NM_005051 | 2.5365 |
| **MARS** | "Methionyl-tRNAsynthetase, cytoplasmic | NM_004990 | 2.4723 |
| **ZSCAN21** | Zinc finger and SCAN domain-containing protein 21 | NM_145914 | 2.4547 |
| **RPL37** | 60S ribosomal protein L37 | XM_294473 | 2.4537 |
| **GTF3C3** | General transcription factor 3C polypeptide 3 | NM_012086 | 2.4097 |
| **JUND** | Transcription factor jun-D. | NM_005354 | 2.3931 |
| **ARS2_HUMAN** | Arsenite-resistance protein 2 | NM_182800;NM_015908 | 2.3764 |
| **ALDH1L2** | "aldehyde dehydrogenase 1 family, member L2 | XM_090294 | 2.3385 |
| **TAF15** | TATA-binding protein-associated factor 2N | NM_003487;NM_139215 | 2.3312 |
| **CXYorf2** | "CDNA FLJ13330 fis, clone OVARC1001802 | NM_025091 | 2.315 |
| **MYST3** | "Histone acetyltransferase MYST3 | NM_006766 | 2.2828 |
| **DMC1** | Meiotic recombination protein DMC1/LIM15 homolog | NM_007068 | 2.2761 |
| **CTBP2** | C-terminal-binding protein 2 (CtBP2) | XM_498499;NM_001329 | 2.2709 |
| **WISP2** | WNT1-inducible-signaling pathway protein 2 precursor | NM_003881 | 2.2695 |
| **SF3B3** | Splicing factor 3B subunit 3 | NM_012426 | 2.2664 |
| **CDK2** | Cell division protein kinase 2 | NM_001798 | 2.2533 |
| **SMARCC1** | **SWI/SNF-related matrix-associated actin-dependent regulator of chromatin subfamily C member 1** | **NM_003074** | **2.2513** |
| **SLC6A5** | Sodium- and chloride-dependent glycine transporter 2 | NM_004211 | 2.2301 |
| **MAGOH** | Protein magonashi homolog. | NM_002370 | 2.2282 |
| **SMR3A** | Submaxillary gland androgen-regulated protein 3 homolog A precursor | XM_496699;NM_012390 | 2.213 |
| **ETV1** | ETS translocation variant 1 | NM_004956 | 2.2077 |
| **SUSD2** | Sushi domain-containing protein 2 precursor | XM_371430 | 2.2023 |
| **ZCCHC11** | Zinc finger CCHC domain-containing protein 11 | NM_001009881;NM_001009882;NM_015269 | 2.1921 |
| **FBXO3** | F-box only protein 3 | NM_012175;NM_033406 | 2.1851 |
| **NLGN1** | Neuroligin-1 precursor | NM_014932 | 2.1797 |
| **SLC25A27** | Mitochondrial uncoupling protein 4 | NM_004277 | 2.1697 |
| **CHEK2** | Serine/threonine-protein kinase Chk2 | NM_007194;XM_375150;NM_001005735;  NM_145862 | 2.1589 |
| **C21orf2** | Uncharacterized protein C21orf2 | NM_004928 | 2.1558 |
| **PWP2** | Periodic tryptophan protein 2 homolog | NM_005049 | 2.1536 |
| **TMC2** | Transmembrane channel-like protein 2 | NM_080751 | 2.1459 |
| **CCNI** | Cyclin-I. | NM_006835;NM_006835 | 2.1229 |
| **ACIN1** | Apoptotic chromatin condensation inducer in the nucleus | NM_014977 | 2.1117 |
| **ZBTB22** | Zinc finger and BTB domain-containing protein 22 | NM_005453 | 2.1102 |
| **C9orf19** | Golgi-associated plant pathogenesis-related protein 1 | NM_022343 | 2.0986 |
| **NGRN** | mesenchymal stem cell protein DSC92 isoform 1 | XM_370904 | 2.0937 |
| **L3MBTL2** | Lethal(3)malignant brain tumor-like 2 protein | NM_001003689;  NM_031488 | 2.091 |
| **BRMS1L** | breast cancer metastasis-suppressor 1-like | NM_032352 | 2.0852 |
| **PAM** | Peptidyl-glycine alpha-amidatingmonooxygenase precursor | NM_138766;NM_000919;NM_138821;NM_138822 | 2.0844 |
| **DIAPH1** | Protein diaphanous homolog 1 | NM_005219 | 2.0765 |
| **GPR120** | G-protein coupled receptor 120 | NM_181745 | 2.0682 |
| **NDEL1** | **Nuclear distribution protein nudE-like 1** | **NM_030808** | **2.0676** |
| **SMC1A** | **Structural maintenance of chromosomes protein 1A** | **NM_006306** | **2.0617** |
| **PKN2** | Serine/threonine-protein kinase N2 | NM_006256 | 2.061 |
| **SULT1C2** | Sulfotransferase 1C2 | NM_006588 | 2.0414 |
| **C5orf15** | Keratinocytes-associated transmembrane protein 2 precursor | NM_020199 | 2.0333 |
| **C20orf10** | TP53-target gene 5 protein (TP53-inducible gene 5 protein) | NM_014477 | 2.032 |
| **VIT** | Vitrin precursor | NM_053276 | 2.0281 |
| **CDC42SE2** | CDC42 small effector 2 | NM_020240 | 2.0198 |
| **FAM92B** | "family with sequence similarity 92, member B, mRNA | NM_198491 | 2.0129 |
| **KIAA0174** | putative MAPK activating protein PM28 | NM_014761 | 2.009 |
| **ZNF410** | Zinc finger protein 410 | NM_021188 | 2.0039 |
| **KIAA1212** | **Hook-related protein 1** | **NM_018084** | **1.6011** |
