# Supplementary material for: Global Transcriptomic Analysis of Human Neuroblastoma Cells in Response to Enterovirus Type 71 Infection
Source: PLoS One. 2013 Jul 5;8(7):e65948. doi: 10.1371/journal.pone.0065948 (PMC3702535; doi:10.1371/journal.pone.0065948)
Supplement: Table S2 — Down-regulated genes in EV71-infected SH-SY5Y cells. (DOC) [file pone.0065948.s002.doc]

**Table S2.Down-regulated genes in EV71-infected SH-SY5Y cells.**

| **Symbol** | **Description** | **Gene Bank** | **Fold change** |
| --- | --- | --- | --- |
| **AMOTL2** | Angiomotin-like protein 2 | NM_016201 | 0.6628 |
| **PRKAA2** | 5'-AMP-activated protein kinase catalytic subunit alpha-2 | NM_006252 | 0.5189 |
| **SUPT3H** | Transcription initiation protein SPT3 homolog | NM_181356 | 0.4989 |
| **KCTD5** | BTB/POZ domain-containing protein KCTD5 | NM_018992 | 0.4981 |
| **JUB** | "jub, ajuba homolog isoform 2 | NM_032876;NM_198086 | 0.4961 |
| **CD28** | T-cell-specific surface glycoprotein CD28 precursor | NM_006139 | 0.4955 |
| **CSMD2** | CUB and sushi domain-containing protein 2 | NM_052896 | 0.4924 |
| **CSF3R** | Granulocyte colony-stimulating factor receptor precursor | NM_156038;NM_000760;NM_172313;NM_156039 | 0.4919 |
| **NCOR2** | "Nuclear receptor corepressor 2 | NM_006312 | 0.4909 |
| **OCA2** | P protein (Melanocyte-specific transporter protein) | NM_000275 | 0.4901 |
| **ASH1L** | Probable histone-lysine N-methyltransferase ASH1L (EC 2.1.1.43) | NM_018489 | 0.4887 |
| **SPHK2** | Sphingosine kinase 2 | NM_020126 | 0.4834 |
| **TPM3** | Tropomyosin alpha-3 chain | NM_152263 | 0.4791 |
| **ZNF155** | Zinc finger protein 155 | NM_198089;NM_003445 | 0.476 |
| **GABPB2** | GA-binding protein beta chain (GABP subunit beta-2) | NM_002041;NM_181427;NM_016655 | 0.4726 |
| **CYP4A11** | Cytochrome P450 4A11 precursor | NM_000778 | 0.4709 |
| **LRTM1** | leucine-rich repeats and transmembrane domains 1 | NM_020678 | 0.469 |
| **TADA3L** | Transcriptional adapter 3-like (ADA3-like protein) | NM_133480;NM_133481 | 0.4677 |
| **ZNF435** | Zinc finger protein 435 | NM_025231 | 0.4654 |
| **GCC2** | Ran-binding protein 2-like 4 | NM_181453 | 0.4652 |
| **GFRA2** | GDNF family receptor alpha-2 precursor | NM_001495 | 0.4616 |
| **RGS6** | Regulator of G-protein signaling 6 | NM_004296 | 0.4603 |
| **LIG1** | DNA ligase 1 | NM_000234 | 0.4593 |
| **SYNJ2** | "Synaptojanin-2 | NM_003898 | 0.4585 |
| **P2RY14** | P2Y purinoceptor 14 (P2Y14) | NM_014879 | 0.4565 |
| **CCL28** | Small inducible cytokine A28 precursor | NM_019846;NM_148672 | 0.4524 |
| **VPS36** | Vacuolar protein sorting-associated protein 36 | NM_016075 | 0.4497 |
| **MFSD3** | Major facilitator superfamily domain-containing protein 3 | NM_138431 | 0.4484 |
| **FNDC5** | fibronectin type III domain containing 5 | NM_153756 | 0.4479 |
| **IRAK1** | Interleukin-1 receptor-associated kinase 1 | NM_001569 | 0.4478 |
| **TGM3** | Protein-glutamine gamma-glutamyltransferase E precursor | NM_003245 | 0.443 |
| **TBX21** | T-box transcription factor TBX21 | NM_013351 | 0.4429 |
| **CSF3** | Granulocyte colony-stimulating factor precursor | NM_000759;NM_172220;  NM_172219 | 0.4421 |
| **TPM1** | Tropomyosin 1 alpha chain | NM_000366 | 0.4384 |
| **CYR61** | "Protein CYR61 precursor | NM_001554 | 0.436 |
| **SOX9** | Transcription factor SOX-9 | NM_000346 | 0.4349 |
| **LILRB3** | Leukocyte immunoglobulin-like receptor subfamily B member 3 precursor | NM_006864 | 0.4313 |
| **FAM43A** | Protein FAM43A | NM_153690 | 0.426 |
| **MUC13** | Mucin-13 precursor | NM_033049 | 0.4193 |
| **TNFSF13B** | Tumor necrosis factor ligand superfamily member 13B | NM_006573 | 0.4181 |
| **LGALS14** | Placental protein 13-like | NM_203471;NM_020129 | 0.4095 |
| **FGD3** | "FYVE, RhoGEF and PH domain-containing protein 3 | NM_033086 | 0.4082 |
| **ZNF691** | Zinc finger protein 691 | NM_015911 | 0.4064 |
| **GRP** | Gastrin-releasing peptide precursor | NM_002091 | 0.4036 |
| **SYN2** | Synapsin-2 (Synapsin II) | NM_133625;NM_003178 | 0.4021 |
| **ACN9** | "ACN9 protein homolog, mitochondrial precursor | NM_020186 | 0.3971 |
| **HLTF** | SWI/SNF-related matrix-associated actin-dependent regulator of chromatin subfamily A member 3 | NM_003071;NM_139048 | 0.3936 |
| **TSPAN12** | Tetraspanin-12 | NM_012338 | 0.3929 |
| **BCAN** | Brevican core protein precursor | NM_021948 | 0.3892 |
| **CD38** | ADP-ribosylcyclase 1 | NM_001775 | 0.389 |
| **CA5A** | "Carbonic anhydrase 5A, mitochondrial precursor | NM_001739 | 0.3875 |
| **CDKL1** | Cyclin-dependent kinase-like 1 | NM_004196 | 0.3819 |
| **HIF1AN** | Hypoxia-inducible factor 1 alpha inhibitor | NM_017902 | 0.379 |
| **ADAMTS2** | ADAMTS-2 precursor | NM_021599;NM_014244 | 0.365 |
| **TNP1** | Spermatid nuclear transition protein 1 | NM_003284 | 0.3429 |
| **JSRP1** | junctional sarcoplasmic reticulum protein 1 | NM_144616 | 0.3413 |
| **CD1C** | T-cell surface glycoprotein CD1c precursor | NM_001765 | 0.3318 |
| **LAT** | Linker for activation of T-cells family member 1 | NM_032038 | 0.3308 |
| **DENND3** | DENN/MADD domain containing 3 | NM_014957 | 0.3293 |
| **TBX19** | "T-box transcription factor TBX19 | NM_199344 | 0.3292 |
| **ABCC3** | Canalicularmultispecific organic anion transporter 2 | NM_020038;NM_003786;  NM_020037 | 0.3218 |
| **C9orf61** | Uncharacterized protein C9orf61 | NM_004816 | 0.3186 |
| **DPYD** | Dihydropyrimidine dehydrogenase [NADP+] precursor | NM_000110 | 0.3116 |
| **RING1** | E3 ubiquitin-protein ligase RING1 | NM_002931 | 0.3068 |
| **STRA6** | stimulated by retinoic acid gene 6 homolog | NM_022369 | 0.3048 |
| **FGFR1OP** | C-C chemokine receptor type 6 | NM_194429;NM_007045 | 0.2959 |
| **HSPA2** | Heat shock-related 70 kDa protein 2 | NM_021979 | 0.2944 |
| **CLCN5** | Chloride channel protein 5 | NM_000084 | 0.2922 |
| **ADAMDEC1** | ADAM DEC1 precursor | NM_014479 | 0.2883 |
| **HTR2B** | 5-hydroxytryptamine 2B receptor | NM_000867 | 0.276 |
| **C4orf6** | Uncharacterized protein C4orf6 | NM_005750 | 0.2755 |
| **WIPF1** | WAS/WASL interacting protein family member 1 | NM_003387 | 0.2703 |
| **TGFBR1** | TGF-beta receptor type-1 precursor | NM_004612 | 0.261 |
| **KCNG2** | Potassium voltage-gated channel subfamily G member 2 | NM_012283 | 0.2555 |
| **CPS1** | "Carbamoyl-phosphate synthase [ammonia], mitochondrial precursor | NM_001875 | 0.2543 |
| **P2RY8** | P2Y purinoceptor 8 (P2Y8) | NM_178129 | 0.2348 |
| **TERF2IP** | Telomeric repeat-binding factor 2-interacting protein 1 | NM_018975 | 0.2344 |
| **IFNAR1** | Interferon-alpha/beta receptor alpha chain precursor (IFN-alpha-REC) | NM_000629 | 0.2324 |
| **PPBP** | Platelet basic protein precursor | NM_002704 | 0.1699 |
| **TGM4** | Protein-glutamine gamma-glutamyltransferase 4 | NM_003241 | 0.1643 |
| **OR5AT1** | Olfactory receptor 5AT1 | NM_001001966 | 0.1507 |
| **SOX10** | Transcription factor SOX-10 | NM_006941 | 0.1297 |
| **COX7B2** | "Cytochrome c oxidase polypeptide VIIb2, mitochondrial precursor | NM_130902 | 0.1241 |
| **TCN1** | Transcobalamin-1 precursor | NM_001062 | 0.0947 |
| **RBM35A** | RNA-binding protein 35A | NM_017697 | 0.0923 |
| **NEK4** | Serine/threonine-protein kinase Nek4 | NM_003157 | 0.0823 |
| **UBE2V1** | Ubiquitin-conjugating enzyme E2 variant 1 | NM_199144;NM_021988;NM_199203;NM_022442;  NM_003349 | 0.072 |
